# Reduction of kinesin I heavy chain decreases tau hyperphosphorylation, aggregation, and memory impairment in Alzheimer’s disease and tauopathy models

**DOI:** 10.3389/fmolb.2022.1050768

**Published:** 2022-10-25

**Authors:** Karthikeyan Selvarasu, Abhay Kumar Singh, Ashok Iyaswamy, Sravan Gopalkrishnashetty Sreenivasmurthy, Senthilkumar Krishnamoorthi, Amal Kanti Bera, Jian-Dong Huang, Siva Sundara Kumar Durairajan

**Affiliations:** ^1^ Molecular Mycology and Neurodegenerative Disease Research Laboratory, Department of Microbiology, Central University of Tamil Nadu, Thiruvarur, India; ^2^ Mr. and Mrs. Ko Chi-Ming Centre for Parkinson’s Disease Research, School of Chinese Medicine, Hong Kong Baptist University, Hong Kong SAR, China; ^3^ Department of Neurology, The University of Texas Medical Branch, Galveston, TX, United States; ^4^ Centre for Trans-Disciplinary Research, Department of Pharmacology, Saveetha Dental College and Hospitals, Chennai, India; ^5^ Department of Biotechnology, Bhupat and Jyoti Mehta School of Biosciences, Indian Institute of Technology Madras, Chennai, India; ^6^ School of Biomedical Sciences, Li Ka Shing Faculty of Medicine, The University of Hong Kong, Hong Kong SAR, China

**Keywords:** Alzheheimer’s disease, tau pathology, kinesin I, KIF5B, P301S tau mice

## Abstract

Many neurodegenerative diseases, such as Alzheimer’s disease (AD) and frontotemporal dementia with Parkinsonism linked to chromosome 17, are characterized by tau pathology. Numerous motor proteins, many of which are involved in synaptic transmission, mediate transport in neurons. Dysfunction in motor protein-mediated neuronal transport mechanisms occurs in several neurodegenerative disorders but remains understudied in AD. Kinesins are the most important molecular motor proteins required for microtubule-dependent transport in neurons, and kinesin-1 is crucial for neuronal transport among all kinesins. Although kinesin-1 is required for normal neuronal functions, the dysfunction of these motor domains leading to neurodegenerative diseases is not fully understood. Here, we reported that the kinesin-I heavy chain (KIF5B), a key molecular motor protein, is involved in tau homeostasis in AD cells and animal models. We found that the levels of KIF5B in P301S tau mice are high. We also found that the knockdown and knockout (KO) of KIFf5B significantly decreased the tau stability, and overexpression of KIF5B in KIF5B-KO cells significantly increased the expression of phosphorylated and total tau levels. This suggested that KIF5B might prevent tau accumulation. By conducting experiments on P301S tau mice, we showed that partially reducing KIF5B levels can reduce hyperphosphorylation of the human tau protein, formation of insoluble aggregates, and memory impairment. Collectively, our results suggested that decreasing KIF5B levels is sufficient to prevent and/or slow down abnormal tau behavior of AD and other tauopathies.

## Introduction

Alzheimer’s disease (AD) is an intricate, progressive, and debilitating neurodegenerative disease marked by gradual memory loss. It is characterized by the formation of β-amyloid (Aβ) plaques in and around neurons and intraneuronal neurofibrillary tangles (NFTs), which result in neuronal death and memory loss. The NFTs consist of microtubule-associated protein (MAP) tau that has been hyperphosphorylated on several Ser/Thr residues (Grundke-Iqbal et al., 1986). Tau is a neuronal MAP that decorates and stabilizes microtubules in the axon where it exhibits a proximo-distal gradient during development (Kempf et al., 1996). Since tau is a phosphoprotein (phospho tau), it is moderately phosphorylated in the normal human brain. However, it is fourfold hyperphosphorylated and polymerized into paired helical filaments/NFTs in the AD brain (Iqbal et al., 2018). Tau pathology also indicates many neurodegenerative diseases, such as frontotemporal dementia with Parkinsonism linked to chromosome 17 (FTDP-17), Pick disease, corticobasal degeneration and progressive supranuclear palsy (Sexton et al., 2022). Neurodegenerative diseases with intracellular Tau filamentous inclusions are generally denoted as tauopathies (Wang and Mandelkow, 2016; Götz et al., 2019). They are accompanied by synaptic failure, transport defects, protein aggregation, and the loss of neurons (Higuchi et al., 2002; Ballatore et al., 2007; De Vos et al., 2008). Neuronal dysfunction and the progression of AD are closely associated with NFT levels (Perrin et al., 2009), and the severity and development of Tau pathology are associated with the Braak stages (Braak and Braak, 1991). The presence of FTDP-17 mutations has provided further evidence for the significance of tau protein in neurodegeneration (Frost et al., 2015). Although the clinical symptoms of tauopathies are known to coincide with early impairment in synaptic function and the neuritic network in the course of the disease, the mechanisms driving these pathogenic events remain unknown.

Axonal transport is crucial in long polarized neurons for delivering proteins, vesicles, and organelles to enable synaptic activity and is facilitated by the tau protein (Goldstein, 2003). Several studies have shown that axonal transport can be impaired by pathogenic forms of tau, including oligomeric tau; this explains why tauopathies cause axonal degeneration (Higuchi et al., 2005; Götz et al., 2006; Cox et al., 2016; Swanson et al., 2017). Molecular motors like kinesin and dynein are responsible for transporting cargoes along microtubules in either the anterograde or the retrograde direction, respectively. The overexpression of tau can interfere with kinesin-dependent transport, which in turn can interfere with the axonal delivery of cargos and proteins (Stamer et al., 2002). The amount of tau attached to the microtubules can modulate kinesin and dynein activities variably (Dixit et al., 2008). The microtubule-binding domain of tau suppresses motor activity, and tau aggregation impairs kinesin-1-mediated trafficking by preventing kinesin-1 from binding to microtubules (Dixit et al., 2008). Additionally, tau phosphorylation can modulate its connections to motor proteins indicating that signaling dysregulation can result in tau mislocalization (Cuchillo-Ibanez et al., 2008).

Multiple motor proteins, most of which are involved in synaptic transmission, mediate intracellular transport in neurons (Hirokawa and Takemura, 2005). Kinesins are the essential molecular motor proteins in neurons and are required for microtubule-dependent transport. Kinesin-1 plays a particularly important role in neuronal transport. Mutations in specific conventional kinesins or cytoplasmic dynein subunits can lead to the dying-back degeneration of neurons, demonstrating the reliance of axons on appropriate anterograde and retrograde transport (Morfini et al., 2009). The dysregulated expression of kinesin superfamily proteins (KIFs) has been studied in several neurodegenerative diseases but less studied in AD (Ittner et al., 2008; Kanaan et al., 2013). Kinesin-1 is a tetramer complex consisting of two heavy and two light chains. Kinesin heavy chain [KHC, kinesin superfamily 5 s (KIF5s)] proteins are encoded by kinesin-1 family genes including KIF5A, KIF5B, and KIF5C. KIF5 binds to the kinesin light chain (KLC) *via* the NH_2_ terminal region, whereas KLC binds to the cargo *via* the COOH terminal region (Rahman et al., 1998). KIF5B consists of three domains: the head domain drives ATP-powered anterograde transport along microtubules, the stalk domain aids in the dimerization of the heavy chains, and the tail domain regulates cargo binding (Hirokawa et al., 2010). Additionally, dysfunctional kinesin-1 is associated with numerous neurodegenerative diseases. For example, lower levels of KIFs were found in the early stages of Parkinson’s disease (PD) before the alteration of dopaminergic markers (Stokin et al., 2005). Also, KIF5B-containing large vesicles, an early characteristic of axonal transport dysfunction, were identified in the post-mortem brain of AD patients (Stokin et al., 2005). High levels of expression were recorded for genes belonging to the kinesin-1 family in people with AD, leading researchers to speculate that an increase in the levels of kinesin-1 isoform expression might hasten neuronal dysregulation in AD patients (Kreft et al., 2014; Hares et al., 2017). A study on Kinesin 1 light chain (KLC)-deficient transgenic Tau mice revealed that defects in axonal transport might lead to aberrant hyperphosphorylation of the tau protein. Tau accumulation and tau-dependent neurodegeneration can occur through axonal stress kinase pathways (Falzone et al., 2010). The depletion of KIF5B is neuroprotective in cerebral ischemia preconditioning (a stroke model) in KIF5B heterozygous knockout mice (Lin et al., 2019). This occurs due to the regulation of calcium influx from extrasynaptic NMDARs and the protection of neurons against NMDA-induced excitotoxicity and ischemia-evoked neurodegeneration. These findings suggest that the inhibition of kinesin-1 might delay neurodegeneration.

To test the hypothesis that the reduction of KIF5B alone can decrease tau accumulation, neurodegeneration, and memory impairment, we partially reduced the levels of KIF5B in P301S tau mutant mice (P301S), a mouse model of AD and FTPD-17, which develops extensive tau pathology throughout the nervous system with high accumulation of phospho tau filaments resembling those of human tauopathies (Allen et al., 2002). We found that an aging-associated decrease in the level of phospho tau accumulation in P301S mice, which were partially lacking the KIF5B protein (P301S^+/−^,KIF5B^+/−^), led to the amelioration of cognitive dysfunction. Additionally, we investigated the functions of KIF5B in regulating phospho tau degradation in cell models. Our results suggested that KIF5B influences tau stability and facilitates tau degradation. The underlying mechanisms of KIF5B-mediated reduction in insoluble tau need to be further investigated. Our *in vivo* findings highlight the importance of KIF5B, the heavy chain of kinesin 1, in regulating Tau abnormalities and tau-related memory deficits in several tau-related neurodegenerative disorders, including AD.

## Materials and methods

### Reagents, plasmids, siRNAs, and antibodies

The materials required for cell culture, including DMEM/F12, DMEM, trypsin-EDTA, glutamine, blasticidin, fetal bovine serum, ECL chemiluminescence detection kits, and Roche’s protease and phosphatase inhibitor cocktail tablets, were purchased from ThermoFisher Scientific (MA, United States). Dharmacon’s Smartpool of 4 small interfering RNA (siRNA), pre-designed to knockdown KIF5B, and Horizon Discovery’s HAP1-wild type (HAP1-WT), a human near-haploid cell line derived from the chronic myelogenous leukemia cell line KBM-7, and HAP1-KIF5B knockout (HAP1-KO) cell lines were purchased from PerkinElmer Ltd. (MA, United States). The antibodies used in this study are as follows: KIF5s polyclonal rabbit antibodies (KIF5A, KIF5B, and KIF5C; Abcam, MA, United States), phospho tau paired helical filament 1 (PHF1) mouse monoclonal antibody (from Prof. Peter Davies), phospho tau monoclonal antibodies (AT8, AT100, and AT180; ThermoFisher Scientific), total tau mouse monoclonal antibodies (Tau-5 and HT7; ThermoFisher Scientific), KLC polyclonal antibody (Santa Cruz, United States), LC3B rabbit polyclonal antibody (Novus Biologicals, CO, United States), amyloid precursor protein (APP) C-terminal rabbit polyclonal antibody (Thermo Scientific), MAP2 rabbit monoclonal antibody (Cell Signaling, MA, United States) and β-actin (ACTB) mouse monoclonal (Cell Signaling). The horse-radish peroxidase (HRP) conjugated AffiniPure goat anti-Mouse IgG (H + L) and goat anti-rabbit IgG (H + L) secondary antibodies were purchased from Jackson ImmunoResearch Inc. (PA, United States). The pCMV-full length human KIF5B was purchased from Origene, (MD, United States). The pRK5-EGFP-Tau P301L (Tau-P301L) and EGFP-Tau WT plasmids (Tau-WT) were gifts from Prof. Karen Hsiao Ashe, Department of Neurology, University of Minnesota, and the plasmids were obtained through Addgene (MA, United States). The transfecting reagents such as Lipofectamine 2000 and Lipofectamine RNAmax were purchased from ThermoFisher Scientific. All chemical reagents were of analytical grade and purchased from Sigma Chemicals (MO, United States) unless otherwise indicated.

### Cell culture

The SHSY5Y neuroblastoma cells stably transfected with the human Tau P301L mutant (SHSY5Y-Tau-P301L) were maintained in DMEM-F12 with 10% FBS containing 1% penicillin and streptomycin and 3.3 μg/ml blasticidin as described in another study (Ke et al., 2012). HAP1-WT and HAP1-KO cells were cultured in a complete DMEM medium cultured following the manufacturer’s instructions.

### Knockdown of KIF5B

The KIF5B siRNA is a target-specific Smartpool siRNA designed to knock down the expression of the KIF5B gene. Briefly, the SHSY5Y-Tau-P301L cells were seeded in 12-well plates at 50% confluency. Then, the different concentrations of the KIF5B siRNA or the non-targeting siRNA were mixed with Lipofectamine RNAimax and added to respective wells. The cells were incubated for 48 h toKD the KIF5B gene.

### Knockout of KIF5B and tau stability

The HAP1-WT or HAP1-KO cells were transfected with the Tau-WT or Tau-P301L or empty plasmid using Lipofectamine 2000, following the manufacturer’s protocol. The overexpression of human KIF5B was performed in HAP1-KO cells in presence of Tau-WT or Tau-P301L plasmids and the level of PHF1, Tau-5 and KIF5B and ACTB were examined by Western blotting. For confocal imaging, HAP1-WT and HAP-KO cells were cultured into a 24-well plate containing coverslips and transfected with the indicated EGFP plasmids and the images of the EGFP for Tau-WT and Tau-P301L expression were captured using a Spinning Disc Confocal microscope (PerkinElmer) with a sequential acquisition setting at 1512 × 1512 pixels resolution. For tau stability assay, both cell lines were transfected with Tau-P301L treated with cycloheximide (CHX, 20 μg/ml) and cells were collected and lysed at different time points.

### Animal experiments

All animal research, including breeding, colony maintenance, and behavioural assessments, were conducted in the University of Hong Kong’s AAALC-accredited laboratory animal unit). The procedures were approved by the Committee on the Use of Live Animals for Teaching and Research (CULATR) (Ref #: 890–04, 1663–08 and #3399-14) at the University of Hong Kong (HKU). The homozygous P301S tau transgenic mice (Allen et al., 2002) were gifted by Prof. Michael Goedert from the MRC Laboratory of Molecular Biology, University of Cambridge, United Kingdom. All mice were housed in a specific pathogen-free facility at LAU, HKU, under a 12 h/12 h light/dark cycle, and provided with food and water. The heterozygous KIF5B knockout (KIF5B^+/−^) mouse line was generated by Prof. Huang’s group (Ni et al., 2018; Lin et al., 2019). The KIF5B^+/−^ mice were crossed with P301S mice to obtain the experimental cohort of P301S^+/−^ and P301S^+/−^,KIF5B^+/−^ in C57B6 background The genotype of all animals was confirmed by polymerase chain reaction (PCR). Genomic DNA was extracted from tail biopsies using the Qiagen genomic DNA extraction kit and processed for PCR genotyping (Ni et al., 2018). All animals were maintained and bred following the guidelines of CULATR. The mice were housed in groups of three to five in cages with a 12 h/12 h light/dark cycle with free access to food and water.

### Open field

To evaluate the locomotor, exploratory, and anxious behaviour of P301S^+/−^, P301S^+/−^,KIF5B^+/−^ and C57B6J (WT) mice in a novel environment, an open field test was carried out as described in our previous studies (Durairajan et al., 2012; Iyaswamy et al., 2021). Mice were placed in a plexiglass box (25 × 25 × 40 cm^3^) and permitted to freely explore the field for a period of 5 minutes. A square 10 cm^2^ in size was chosen as the centre area in the centre of the open field. Movement patterns, time spent in the centre and margin, and distance travelled were all tracked and measured using the Ethovision video tracking system (Noldus Information Technology, United States) and Ethovision XT software, respectively. The distance travelled by the mice was used as a measure of locomotion, while time spent in the central square was used to measure anxiety-like behaviour.

### Rotarod

The rotarod test was conducted to evaluate motor function behavior (Harvard Apparatus, SEDACOM v2.0.000). About 30 min before the test, P301S^+/-^, P301S^+/−^,KIF5B^+/−^ and WT mice were brought into the lab. The mice were trained on a rotating shaft at 4, 8, and 12 rpm on days 1, 2, and 3, respectively. Three trials were performed every day, and the amount of time spent on the rotating rod was measured. On day 4, an acceleration mode (4–40 rpm in 5 min) was used, and the amount of time spent by the mice on the rotarod was recorded.

### Contextual fear conditioning

The contextual fear conditioning (CFC) test was performed in P301S^+/−^, P301S^+/−^,KIF5B^+/−^ and WT mice to assess fear memory consolidation based on the protocol described in our previous studies (Song et al., 2020; Sreenivasmurthy et al., 2022). The experimental setup consisted of two soundproof chambers, each with an electric grid on the surface. A speaker was installed in each chamber to provide a cue tone along with continuous white noise (40 units) and white light (40 lux). On the first day of training, the mice were introduced to the chamber and allowed to explore the new environment for 2.5 min. Then, each mouse experienced foot shock cycles thrice in a row, each of which began with a cue tone (1,500 Hz) for 28 s and ended with a foot shock (30.0 mA) for 2 s. On the second day, the mice were allowed to explore the grid for 3 min before receiving a cue tone for 30 s without a foot shock. The movement time and the freezing time of each mouse were monitored and recorded by the ANY maze tracking software (Ugo Basile, Itay).

### Tau extraction

At the end of the behavioral experiments, the mice were sacrificed by CO_2_ euthanasia. The brain tissues of P301S^+/−^ and P301S^+/−^,KIF5B^+/−^ mice were dissected sagitally into two equal hemispheres and one-half of the samples were processed for immunohistochemical analysis, while the other hemispheres were snap-frozen for biochemical studies on tau. The soluble and insoluble tau fractionation was performed based on our previous study (Myeku et al., 2016; Iyaswamy et al., 2021). The brain samples were homogenized in RIPA buffer amended with a protease and phosphatase inhibitor cocktail, and the brain lysate was centrifuged at 21,130 g for 30 min to separate proteins. The RIPA-soluble fraction was incubated with 1% sarkosyl for 2 h at room temperature and ultracentrifuged at 100,000 g for 1 h to separate into sarkosyl soluble and insoluble (pellet) fractions. The pellet was then resuspended in 20 µL of Tris-EDTA buffer. The sarkosyl soluble and insoluble fractions were used for the immunoblotting detection of human total tau (HT7) and phospho tau epitopes (AT8, AT180, AT100 and PHF1). The RIPA fraction was analyzed by performing western blot analyses to determine the levels of KIF5A, KIF5B, KIF5C, KLC, Fl-APP, MAP2, LC3, and ACTB proteins.

### Immunohistochemistry

The brain tissues from the left hemisphere of P301S^+/−^, P301S^+/−^, KIF5Band WT mice were fixed with paraformaldehyde for 48 h and processed in 30% sucrose for cryopreservation. Then, the tissues were embedded in an optimum cutting temperature compound and used for immunohistochemistry (IHC) assays as described in our previous study (Duarirajan et al., 2012). Three coronal brain sections (per set) of 30 μm with a 120 μm spacing from the anterior, medial, and posterior cortico-hippocampal regions were made using a Shandon Cryotome SME (Ramsey; MN, United States) maintained at −20°C. After permeabilization of the section with 0.4% Triton X-100 for 10 min, biotinylated AT8 primary antibodies (1:100) were added overnight and kept at 4°C to probe the phospho tau positive neurons. Staining was performed using a Vectastain-ELITE ABC-HRP kit following the manufacturer’s protocol. The immunostained slices were spread on the glass slides and mounted using a coverslip with an appropriate mounting medium. All images of cortical/hippocampal regions were recorded using an Eclipse 80i fluorescence microscope (Nikon Instruments Inc.). After removing the background and the nonspecific staining level, a threshold optical density was established, which indicated a particular immunoreactive signal. This optical density was then maintained while processing the image, and the region of interest was captured for each section from the anterior, medial, and posterior regions (nine sections per mouse) to evaluate the number of AT8 immune-positive neurons using the Image J (NIH) software.

### Western blot analysis

Immunoblot analysis was performed following the protocol described in our previous study (Iyaswamy et al., 2021). For cell culture experiments, the cells were solubilized using cold RIPA lysis buffer containing protease and phosphatase inhibitor tablets. The cells were sonicated and centrifuged at 14,000 rpm for 15 min. For the biochemical analysis of tau, the RIPA, sarkosyl soluble, and insoluble fractions (equal to 5 and 30 μg of total protein of cell and brain lysate, respectively) were separated on 10%–15% double layered SDS–PAGE gels, and the proteins were blotted onto PVDF membranes. The blotted membranes were blocked with 5% skimmed milk suspended in 0.1% Tween 20 in Tris-buffered saline. The blots were probed with primary antibodies overnight at 4°C with constant shaking to detect the level of PHF1, Tau-5, HT7, PHF-1 AT8, AT180, AT100, MAP2, LC3-II, KIF5A, KIF5B, KIF5C, KLC, and ACTB proteins. After overnight incubation, the blots were washed in TBST and incubated with the corresponding HRP-conjugated secondary antibodies at room temperature. The chemiluminescent signals were captured using an X-ray film in a dark room with an appropriate chemiluminescent substrate kit. The films were scanned, and the relative intensities of the signals were analyzed using the Image J software.

### Statistical analysis

The data collected from the behavioural experiments were analyzed by performing a one-way analysis of variance (ANOVA) with measures of “genotype”, “effect,” and their interactions as fixed factors. Then, a post hoc comparison of the differences between the means of the groups was made by conducting Dunnett’s tests. The IHC data were also analyzed using a one-way and two-way ANOVA followed by Bonferroni post hoc comparisons. For cell culture experiments, a two-paired T-test was performed to analyse the mean difference between the two groups. Statistical significance was determined, and data were graphically represented using the GraphPad Prism 6 Software.

## Results

### Elevated levels of KIF5B in the brains of P301S mice

**FIGURE 1:**
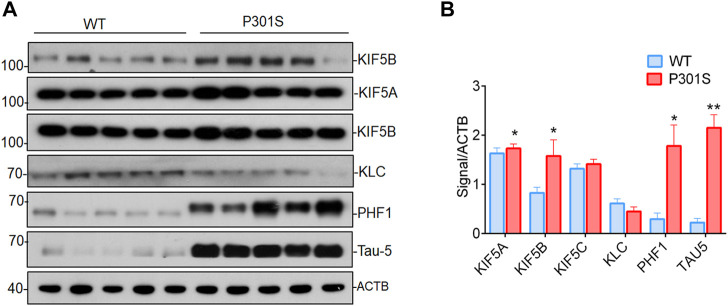
Accumulation of KIF5B, phospho tau and total tau proteins in P301S mice). **(A)** Western blotting was performed to detect the expression of KIF5B, KLC, phospho tau PHF1 and total tau 5 (Tau-5) in the brain lysates of 4-month-old WT and P301S mice; **(B)** Quantitative analysis of immunoblot showed that KIF5B, PHF1 and Tau-5 but not KLC levels are significantly increased in P310S mice (*n* = 5). The bar diagram represents the means of 3 independent experiments; the error bars represent the SEM (mean ± SEM) of 3 independent experiments, *p < 0.05, and **p < 0.01 (WT vs. P301S).

Alzheimer’s disease and other neurodegenerative tauopathies are characterized by the accumulation of hyperphosphorylated tau in cell bodies, which may eventually lead to the formation of neurofibrillary tangles (NFT) (Ballatore et al., 2007). The kinesin-1 superfamily members, such as KIF5A, B, and C, are associated with AD (Kreft et al., 2014; Hares et al., 2017). To determine whether KIF5s or KLC are involved in tau accumulation, we measured KIF5A, KIF5B, KIF5C, KLC, phospho tau, and total tau protein levels in the brains of P301S mice. This mouse model has human tau with the P301S mutation and shows many features of human tauopathies (Allen et al., 2002). The P301S mice had high levels of phospho tau at PHF-1, and total tau, as found in four-month-old mice compared to the WT mice (Figure 1A). This early pathology depended on the presence of the mutant tau protein and was related to the onset of axonopathies in mice. This was probably due to axonal transport defects in the P301S mice (Bull et al., 2012). Among the KIF5s, the protein levels of KIF5B was significantly increased (Figures 1A,B) in the brain lysates of P301S mice compared to WT mice. The KIF5B protein levels, along with phospho tau levels, were significantly higher in the four-month-old P301S Tau mice compared to their levels in the age-controlled WT mice (Figures 1A,B). Slightly lower levels of KLC-1 were also detected in the brain of the P301S Tau mice. These results suggested that KIF5B might play a specific role in this AD animal model.

### Reduction of KIF5B decreases phospho tau levels in SHSY5Y Tau-P301L cells

**FIGURE 2:**
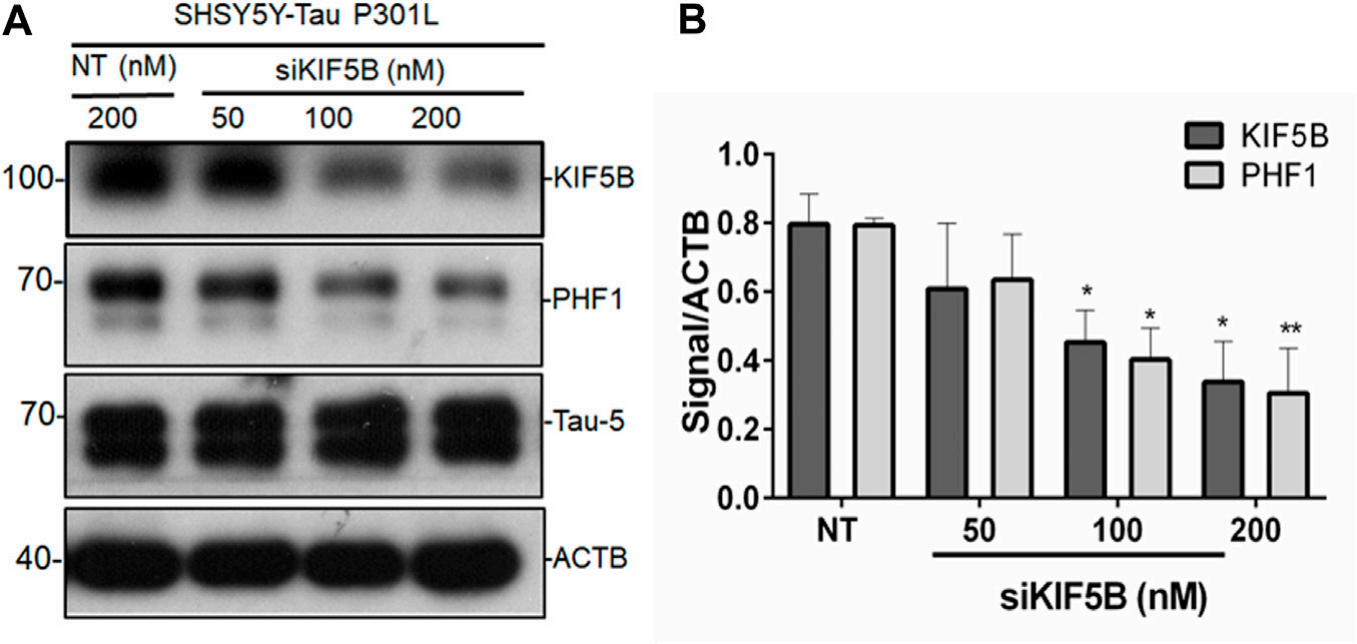
KIF5B negatively regulates phospho tau PHF1. **(A)** The protein level of KIF5B, PHF1 and Tau-5 was measured in western blotting after transfection of SHSY5Y-Tau-P301L cells with KIF5B siRNA or nontargeting (NT) siRNA; ACTB was used as a loading control. **(B)** Quantitative analysis showed that KIF5B knockdown significantly increased KIF5B and PHF1 levels without altering Tau-5. The histogram represents the mean ± SEM of 3 independent experiments; *p < 0.05, ***p < 0.01 significantly different from NT-siRNA control-treated cells.

Since we found that KIF5B levels rise in P301S mice, we hypothesized that KIF5B might regulate tau phosphorylation and aggregation. To establish the regulatory functions of KIF5B in tau phosphorylation, we first knocked down KIF5B in SHSY5Y-Tau- P301L, an extensively used AD cell model (Ke et al., 2012). The depletion of KIF5B for 48 h with varying concentrations of siRNA resulted in a concentration-dependent decrease in KIF5B levels without affecting the neur outgrowth of SHSY5Y-Tau-P301L (Figures 2A,B). We found that KIF5B knockdown significantly reduced the level of phospho tau at PHF1 but did not affect the levels of total tau (Figure 2B).

**FIGURE 3:**
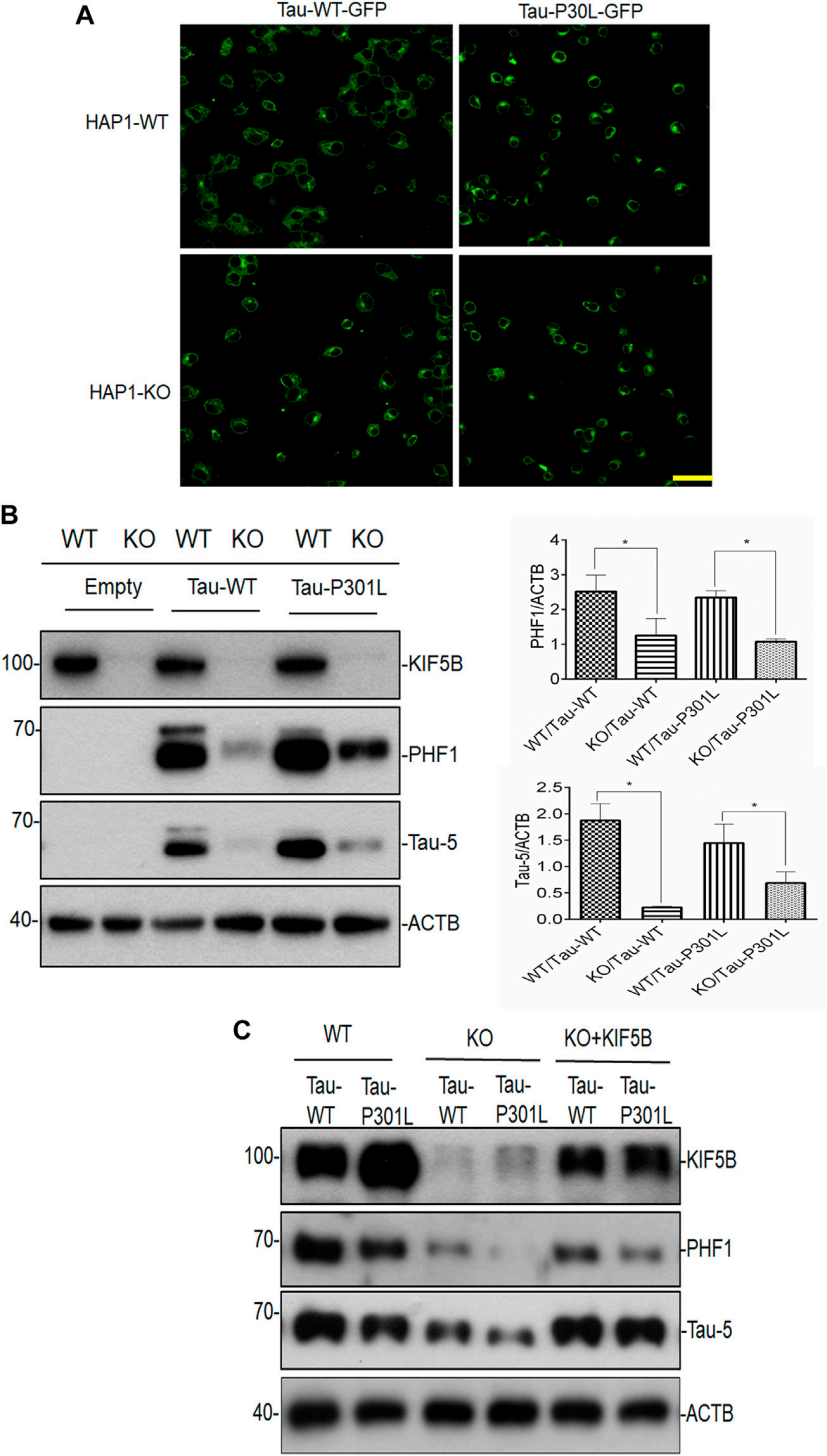
Lack of KIF5B reduces both phospho tau PHF1 and total tau. **(A)** Following transfection of HAP1-WT and HAP1-KO cells with plasmids encoding Tau-WT-GFP or Tau-P301L-GFP or empty plasmid, the fluorescence images were recorded using a confocal laser scanning microscope. Representative confocal images of HAPI-KO transfected cells show a decrease in filamentous tau aggregates, compared with the HAP-WT transfected cells. The scale bar is 20 μm. **(B)** The level of KIF5B, phospho t au PHF1 and total tau Tau-5 was also assessed in the transfected cells by western blotting and the signals of PHF1 and Tau-5 were quantified in the histogram using ACTB as an internal control. Histogram analysis data showed that HAP1KO but not HAP1-WT cells showed reduced PHF1 and Tau-5 levels. The data were represented as mean ± SEM of 3 independent experiments. *p < 0.05 and **p < 0.01. *p < 0.05, ***p < 0.01 significantly different from HAP-WT transfected cells. **(C)** Overexpression of KIF5B in HAP-KO cells increases PHF1 and Tau-5 levels. HAP-KO cells were transfected with Tau-WT or Tau-P301L plasmid in the presence or absence of a KIF5B expression construct (+KIF5B add back). The overexpression of KIF5B significantly reversed the HAP1-KO-mediated decrease in the level of PHF1 and total tau.

Additionally, we examined the amount of phospho tau and total tau in the human near-haploid cell line HAP1- KO and its parental HAP1-WT. Both the CRISPR-Cas9-engineered HAP1-KO and HAP1-WT cell lines were used in the overexpression of tau plasmids The morphology of the HAP1-KO cells was similar to that of the HAP1-WT cells (data not shown). In these cell lines, Tau-P301L construct, a strongly aggregating mutant, and Tau-WT construct were expressed, and their ability to induce tau accumulation was investigated. Since these tau constructs contained the EGFP tag, the level of tau expression was also visualized by capturing the EGFP signal using a confocal laser scanning microscope (Figure 3A). In the HAP1-WT cells, large tau aggregates of filamentous in appearance, were seen clustered in the perinuclear area in cells transfected with both P301L-Tau and Tau-WT constructs (Figure 3A). Additionally, the cells transfected with the Tau-P301L construct showed a greater increase in the appearance of aggregated Tau compared to the Tau-WT transfected cells. In contrast, the HAP1-KIF5B-KO cells had fewer Tau aggregates and filaments than the HAP1-WT cells (Figure 3A). To validate the aforementioned findings, we performed a Western blot analysis of Tau for both transfected cell lines. The phospho tand total tau levels were considerably lower in the HAP1-KIF5B KO cells compared to that in the HAP1-WT cells (Figure 3B). The overexpressed wild-type Tau was removed efficiently in the absence of KIF5B, considering that the rate of its degradation was substantially higher in the KO cells than in the Tau-P301L-expressing cells. To further confirm whether KIF5B was important in regulating phospho and total tau levels, HAP1-WT or HAP1- KO cells were transfected overnight with the Tau-WT or Tau-P301L expression construct, and the KO cells alone were transfected with Tau-WT or Tau-P301L expression construct in the presence or absence of a full length mammalian KIF5B expression construct (+KIF5B add back). The KO-mediated reduction of tau was abrogated by the add back of KIF5B (Figure 3C) These results provided further evidence that KIF5B KO might play a crucial role in phospho tau and overall tau degradation.

### KIF5B knockout affects protein stability of the mutant tau protein

**FIGURE 4:**
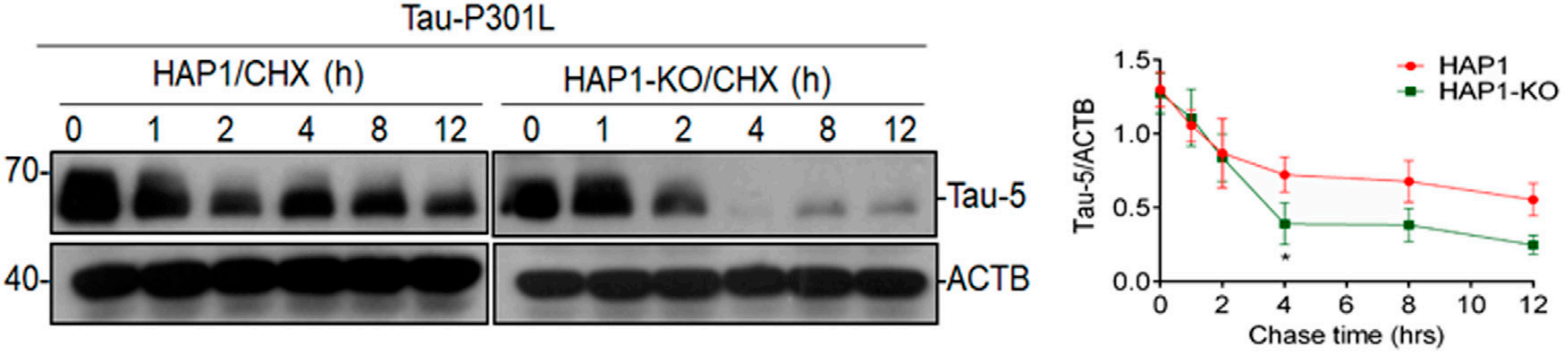
Effect of KIF5B knockout on the protein stability of Tau-P301L Tau mutant. HAP1-WT or HAP1-KO cells were transfected with Tau-P301L plasmid overnight followed by the addition of cycloheximide to stop new protein synthesis and chased for indicated times. Cell lysates from different time points and analyzed by immunoblotting with Tau-5 and ACTB antibodies. Graphical representation of HAP1-WT and HAP1-KO transfected Tau-P301L plasmid with cycloheximide chasing experiment. The levels of Tau-5 was quantified from each timepoint and the data were represented as mean ± SEM of three independent experiments. *p < 0.05 significantly different from HAP-WT transfected cells.

To determine whether KIF5B knockout affects the stability of Tau-P301L proteins, the HAP1-KO and HAP1-WT cells were co-transfected with Tau-P301L mutant expression construct overnight, followed by the addition of cycloheximide to inhibit tau synthesis. Finally, the turnover of tau was determined at various times, as described in our previous study (Yang et al., 2017). The cycloheximide chase analysis showed that the half-life of tau proteins was proportional to the rate at which the tau steady-state levels decreased (Figure 4). In the HAP1-WT cells, tau levels were highly stable but were lower in the HAP1-KO cells. These findings indicated that the degradation of tau in KO cells was higher.

### Reducing KIF5B rescues the phenotypes in a tauopathy mouse model

The finding that KIF5B knockdown or knockout in cell cultures and cultures increased the protein stability of WT tau was consistent with the findings of another study that showed that the reduction of KIF5B can be neuroprotective in the mouse cerebral ischemia preconditioning model, especially by disturbing the interaction of KIF5B and the NMDA receptor subunit GluN2B (Lin et al., 2019). These findings indicated that kinesin-1 and other intracellular transport mechanisms could be targeted to delay neurodegeneration. To further confirm these findings and investigate their neuroprotective importance, we analyzed littermates derived from crosses between P301S mice and KIF5B^+/−^ mice to determine the effect of the reduction of KIF5B on tau-related memory deficits and abnormalities produced by P301S mice.

### Open-field and rotarod tests

**FIGURE 5:**
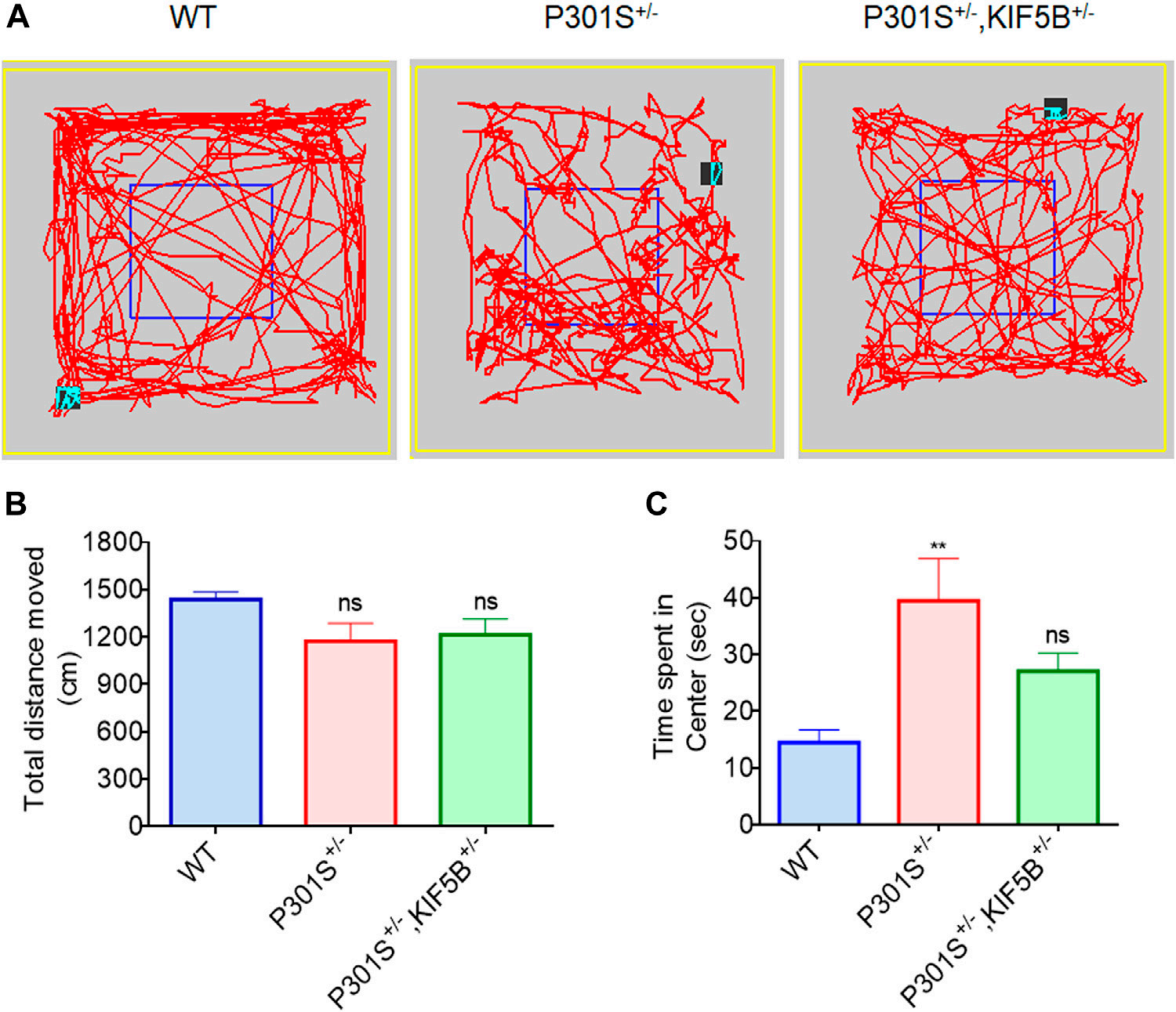
Open field behavioural analysis. **(A)** Representative patterns of exploratory behaviour exhibited by mice in each group. **(B)** Total distance travelled in the open field test. **(C)** Quantification of time spent in the center for each group. Date represent mean ± SEM and WT mice (n=13), P301S+/− (n=14), P301S+/−,KIF5B+/− (n=14). **p < .001 vs. WT; ns > 0.05 vs. WT

**FIGURE 6:**
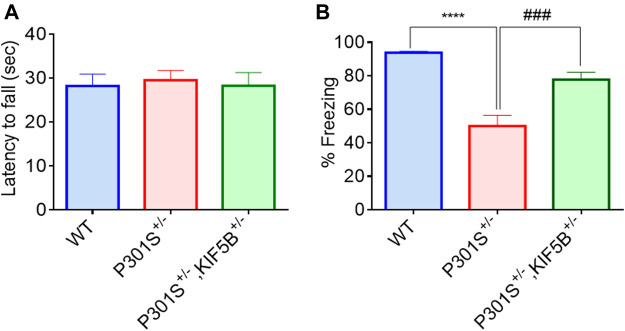
Deficiency of KIF5B ameliorated hippocampal and amygdala-dependent memory deficit in P301S tau mice without influencing their motor function. **(A)** In the rotarod test, the average latency of fall was calculated for WT, P301S+/− and P301S+/−,KIF5B+/− mice at 8 -months old (n = 11−15), and none of the groups show motor dysfunction. **(B)** In the Contextual fear conditioning test, the mean % freezing was quantified in each group and data were presented in mean +SEM. ****p < 0.0001 vs. WT mice, ###p < 0.001 vs. P301S+/− mice, analyzed by one-way ANOVA with post hoc Tukey's Multiple comparison test.

The 5–6-month-old P301S Tau homozygous mice showed typical neurological abnormalities, including muscular weakness, tremors, and severe paralysis/paraparesis (Allen et al., 2002). However, the exploratory behaviour and motor function of P301S^+/−^ and P301S^+/−^,KIF5B^+/−^ heterozygous mice were not evaluated previously. Hence, we first conducted the open field test to assess the exploratory and anxious behaviours of WT, P301S^+/−^ or P301S^+/−^,KIF5B^+/−^ mice in a novel environment. There is no significant difference between WT and P301S^+/−^or P301S^+/−^,KIF5B^+/−^mice in terms of overall movement and distance travelled. (Figures 5 A,B). The open field test also revealed that P301S^+/−^mice spent significantly more time at center compared with the WT mice indicating a higher anxiety level for P301S^+/−^ mice. Notably P301S^+/−^, KIF5B^+/−^ spent less time in the center region than P301S^+/−^ though there is no significant difference between these two transgenic mouse groups (Figures 5 A,C). These results indicate that reduction of KIF5B could slightly reverse anxious behaviour observed in P301S^+/−^mice. We then tested the motor function of P301S^+/−^ and P301S^+/−^,KIF5B^+/−^ mice by conducting a rotarod test. The results showed that the WT and P301S^+/−^ or P301S^+/−^, KIF5B^+/−^ mice did not differ significantly in the latency to fall in the acceleration rotarod test (Figure 6A). These findings suggested that the P301S^+/−^ and P301S^+/−^,KIF5B^+/−^ mice have normal motor functions, indicating that the decrease in KIF5B does not alter the motor function of Tau mice, and thus, these mice can be tested for memory.

### Contextual and cued fear conditioning test

To examine the memory reconsolidation and retention of P301S^+/−^, P301S^+/−^,KIF5B^+/−^ mice and their WT controls, a fear conditioning test was conducted as described in our previous study (Iyaswamy et al., 2021). The mice were habituated for 1 min on a shocking grid, and fear conditioning was performed with three pairings of a 28 s, 5K Hz, 70 dB auditory cue (conditional stimulus), followed by three consecutive 0.5 mA foot shocks (unconditioned stimulus) for 2 s. The inter-trial interval was 20 s. After conditioning for 1 min, the mice were returned to their home cages. For the recall test, the mice were initially placed in a different context for 3 min (pre-tone), and this was followed by cue tone presentation (30 s, 1500 Hz) without foot shock for 3 min (conditional stimulus). The time spent by the mice without moving (freezing time) was recorded. Contextual fear memory formation and remote memory stabilization were measured by determining the freezing index. The evaluation of contextual memory and freezing behavior on the second day showed that the WT mice exhibited longer freezing time than the transgenic mice in response to the changing context and cue tone. In contrast to the P301S^+/−^ mice, the mice in the P301S^+/−^,KIF5B^+/−^ group exhibited a longer duration of freezing and enhanced memory performance (Figure 6B). These results indicated that reducing KIF5B in P301S Tau mice improved their hippocampus-dependent and amygdala-dependent cognitive deficits in fear training.

### Depletion of KIF5B decreases tau pathology in P301S mice

**FIGURE 7:**
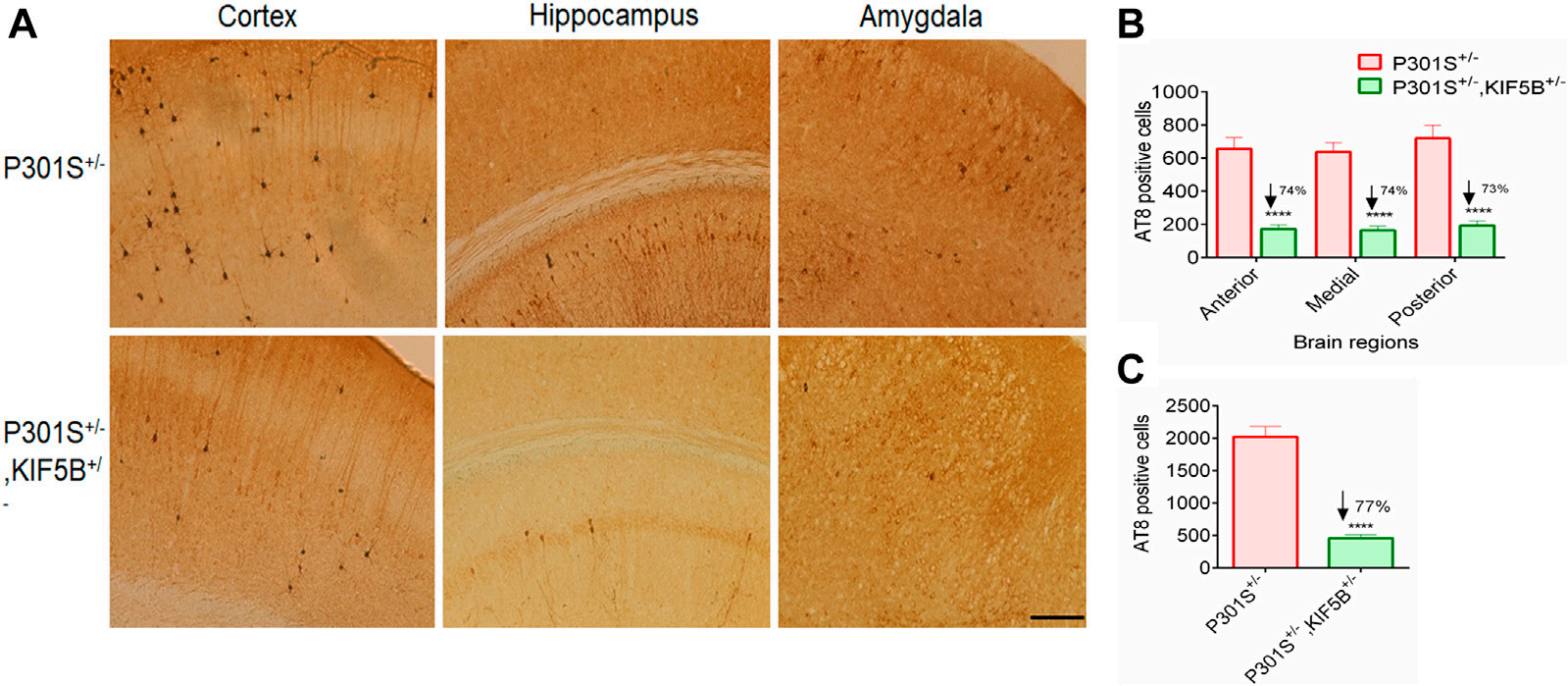
Decreased AT8-positive tau pathology in the brain of KIF5B reduction combined with P301S Tau. **(A)** Immunostaining of AT8 tau in the coronal brain section of P301S^+/−^ and P301S^+/−^,KIF5B^+/−^ **(B,C)** Reduction of area-wise and total count of AT8-positive neurons were shown for all overall half coronal hemisphere of P301S^+/−^,KIF5B^+/−^ mice. Values represent group mean ± SEM, and *n* = 14 mice per group. In **(B)**, ***p < 0.0001 vs. P301S^+/−^ after two-way ANOVA followed by Turkey post hoc analysis. In **(C)**, ****p < 0.0001 vs. P301S^+/−^ after one-way ANOVA followed by Turkey post hoc analysis.

At the end of the behavioral tests, the mice were sacrificed, and their brains were removed for biochemical and immunohistochemical analyses. To determine whether improvement in memory observed in the P301S^+/−^,KIF5B^+/−^ mice was mediated by the attenuation of tau pathology, we performed immunohistochemical staining of the cortico-hippocampal sections for phospho tau from P301S mice with or without reduction in KIF5B, using the AT8 antibody. The epitope of AT8 is located outside the region of internal repeats of microtubule-binding domains and requires the phosphorylation of S202/T205. An examination of the AT8-positive neurons in the P301S^+/−^ and P301S^+/−^,KIF5B^+/−^ mice revealed that heterozygous deletion of KIF5B in the P301S mice significantly reduced the number of AT8-positive neurons in the brain sections of the cortex, amygdala and hippocampus regions (Figure 7A).

Quantification of AT8-positive neurons showed a significantly lower AT8 burden in the P301S^+/−^,KIF5B^+/−^ mice compared to that in the P301S^+/−^ mice. To determine which areas of the brain showed considerable reduction in AT8 immunoreactivity, four coronal sections (per set) from the anterior, medial, and posterior hippocampus were made at an interval of 120 µm on a cryostat at −20°C and a thickness of 30 µm in each region, and the AT8-positive neural plaques were counted. The results of the two-way ANOVA showed a genotype effect [F (2,66) = 435.1; *p* < 0.0001] related to the mean number of AT8-positive neurons in different brain regions (Figure 7B). Post hoc multiple analyses revealed that the mice in the P301S^+/−^, KIF5B^+/−^ group showed a significant reduction in the anterior, medial, and posterior regions of the brain, and there was a 74% reduction in AT8-positive neurons in the anterior, medial, and posterior regions (*p* < 0.0001). There was a 77% reduction in the overall number of AT8-positive neurons in the cortico-hippocampal region of the P301S^+/−^,KIF5B^+/−^ mice compared to that in the P301S^+/−^ mice (Figure 7C). These findings suggested that KIF5B reduction affects the formation of phospho tau in P301S mice.

### Reduction of KIF5B enhances the degradation of phospho tau aggregates

**FIGURE 8:**
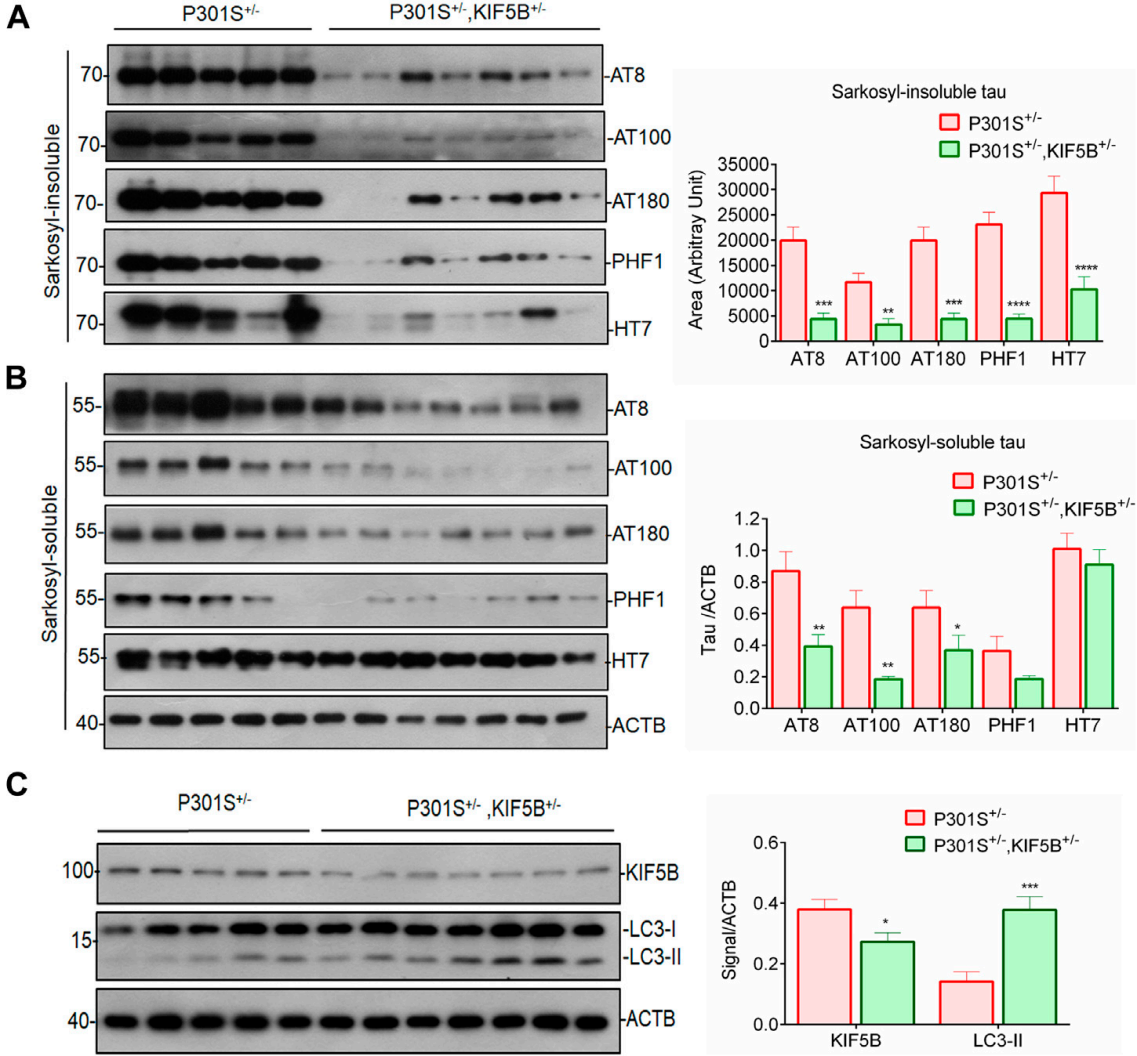
Depletion of KIF5B reduces insoluble tau accumulation in the brain of tau mice. Western blots showed phosphorylated forms of tau (AT8, AT180, AT100, PHF1) and total tau (HT7) in sarkosyl-insoluble **(A)** and insoluble **(B)** fractions after sarkosyl extraction from the whole brain lysates of P301S^+/−^ and P301S^+/−^,KIF5B^+/−^ at 8-month-old mice. **(C)** KIF5B reduction also increased the level of LC3-II protein, an autophagic marker, in the whole brain lysate of P301S^+/−^,KIF5B^+/−^. ACTB was used as an internal control. Values represent group mean ± SEM and *n* = 10–14 mice per group. *p < 0.05, **p < 0.01, ***p < 0.0001, ****p < 0.0001 vs. P301S^+/−^after conducting paired T-test.

Next, by sarkosyl fractioning of mouse brains, we determined whether a reduction in KIF5B levels decreases soluble or insoluble tau species., as. To assess tau pathology in the brains of P301S^+/−^ and P301S^+/−^,KIF5B^+/−^ mice, we used AT8, AT100, AT180, and PHF1 monoclonal antibodies, which are commonly used as diagnostic markers of Alzheimer’s disease. AT8 is diagnosed through the phosphorylation of Se96/Ser404, an epitope that is positioned outside the region of microtubule-binding repeat domains. The epitope AT100 is also located outside RT-14 and requires the phosphorylation of Thr212/Ser214. The AT180 and PHF1 antibodies recognize the phospho tau epitopes at Thr231 and Ser394/Ser404, respectively. In both genotypes, tau proteins with a molecular weight of 64 kDa were predominant in the insoluble fractions compared to the nonpathogenic tau species with a molecular weight of 55 kDa. The P301S^+/−^ heterozygous mice showed excessive accumulation of phospho tau species, which indicated the presence of neurofibrillary tangles (Figure 8A). The soluble and insoluble phospho tau levels were considerably lower in the P301S^+/−^,KIF5B^+/−^ mice compared to that in the P301S^+/−^ mice, and the phospho tau levels further decreased by 3–4-fold due to a decrease in the levels of KIF5B (Figures 8A,B), which was consistent with the findings that KIF5B KD or KO decreased Tau-P301L stability in cell culture experiments (Figures 2, 3). We also assessed the KIF5B protein levels in the P301S^+/−^ and P301S^+/−^,KIF5B^+/−^ mice by immunoblotting and found that the heterozygosity of KIF5B KO mice decreased the level of KIF5B by 42.5 ± 10.7. We also found that the level of the autophagy marker LC3-II protein increased two-fold in the KIF5B KO mice compared to the level of LC3-II protein in the P301S^+/−^ mice (Figure 8C).

The results of our preliminary experiment showed that P301S^+/−^,KIF5B^+/−^ mice always had fewer AT8-positive neurons compared to P301S^+/−^ mice (Supplementary Figure S1A). These results suggested lower levels of hyperphosphorylated tau in neurons with KIF5B reduction. In the biochemical analysis of fractionated tau species, we also found that the level of soluble and insoluble tau at various phospho tau epitopes (AT8 and PHF-1) were significantly lower in the brain of the P301S^+/−^,KIF5B^+/−^ mice (Supplementary Figure S1B) with a two-fold reduction in the level of KIF5B and a two-fold increase in the LC3-II protein levels without a change in the MAP2 and Fl-APP levels (Supplementary Figure S1B). A decrease in the KIF5B levels promoted autophagy, which was partly responsible for the decrease in tau. Based on the difference in the AT8-positive neurons between the groups (P301S^+/−^ and P301S^+/−^,KIF5B^+/−^ mice), the predicted effect size of each group was 2.44. And according to G*power calculations, the sample size of each group was found to be 12 (*p* < 0.05 for 95% power) with 72% reduction in AT8 positive neurons by P301S^+/−^,KIF5B^+/−^. However, we assigned 14–17 mice to each group because some mice might die during experiments.

## Discussion

In AD and other tauopathies, aberrant hyperphosphorylation of tau is an early and critical step, as it can lead to microtubule instability, the accumulation of insoluble tau species, and the formation of neurofibrillary tangles. However, the relationship between Tau dysfunction and neurodegeneration is only partly understood, particularly in the early phases when Tau is mislocalized but not aggregated. Defective axonal transport might be a pathomechanism of AD and other tauopathies, and this was hypothesized based on cellular and transgenic Tau models. One such model is the human P301S tau transgenic mouse strain which, like humans with tauopathies, exhibits widespread tau pathology throughout the nervous system due to the deposition of many tau filaments (Allen et al., 2002). The P301S tau homozygous mice showed abnormally large axons in the optic nerve when they were 5 months old, as well as AT100-positive phospho tau aggregates in axons and degenerating mitochondria and organelles. These findings indicated a continuous degenerative process. The kinesin-1 superfamily members, such as KIF5A, B, and C, are associated with AD (Kreft et al., 2014; Hares et al., 2017). However, these findings regarding the kinesin protein were later challenged by a study showing marked downregulation of both KIF5A and KIF5B proteins in 5XFAD mice (Wang et al., 2019), which suggested that the role of KIF5B in 5XFAD (APP mice) might be mouse model-dependent. Hence, whether KIF5B is a positive or negative regulator in AD models remains unclear. In this study, we found that the level of KIF5B, among the member of the Kinesin 1 heavy chain motor proteins, was higher in brain samples from homozygous P301S Tau mice while the light chain remained unaltered. These results suggested that KIF5B might play a role in the development of AD (Figure 1). The results from this study and recent publications confirmed the abundant accumulation of phospho tau and total tau species in five-month-old mice (Figure 1. The low levels of axonal transport observed in P301S homozygous mice most probably occurred due to the hyperphosphorylation and aggregation of tau, rather than the overexpression of monomeric human mutant tau (Bull et al., 2012). Studies on axonal transport in the ventral roots of mice transgenic for the R406W mutation in tau, and also in the neurons of substantia nigra pars compacta of mice transgenic for the K369I mutation in tau reached the same conclusion. However, deficits in axonal transport were also found in wild-type tau mice and these findings provided new insights into the axonal deficit observed in AD-related tauopathies.

Tau prevents the motor protein kinesin from attaching to microtubules (Stamer et al., 2002). Additionally, a tau gradient was identified along the axon, with the highest levels of tau located proximal to the synapse (Mandell and Bakner, 1996). Mandlekow group and others hypothesized that tau protein hinders axonal transport by preventing kinesin from binding to the microtubules (Ebneth et al., 1998; Mandelkow et al., 2003). Another study hypothesized that tau could reverse dynein directionality by preventing kinesin from attaching to the microtubules (Dixit et al., 2008). However, other results contradicted this hypothesis (LaPointe et al., 2009; McVicker et al., 2011). The role of the dysregulation of kinesin expression in neurodegenerative diseases was investigated; however, the focus was limited to AD and other tauopathies. Correlation-based evidence suggested that the overexpression of the kinesin 1 family occurs in the brain samples of AD patients. Although upregulation of KIFs occurs in AD, whether or not it contributes to neurodegeneration is not known. The role of KIF5B-mediated transport in AD also needs to be determined.

In this study, we found that the depletion of KIF5B prevented memory impairment and tau deposition in P301S^+/−^ transgenic mice by inhibiting tau phosphorylation and tau pathology, thus, playing a protective role. Using the SHSY5Y cell model of Alzheimer’s disease, we found that KIF5B plays a crucial role in regulating phospho tau levels. Although KIF5B knockdown decreased the phosphorylation of PHF1 tau at ser396 (Figures 2, 3), KIF5B did not affect the total tau levels. In the HAP1-KO cell model, we found that KIF5B knockout decreased phospho tau and total tau. We also found that the overexpression of KIF5B increased tau levels in HAP1-KO cells, and this effect depended on the co-expression of wild-type tau and mutant tau. KIF5B significantly affected the protein stability of Tau-P301L, and the effects depended on the absence of KIF5B in P301-Tau tau-transfected cells (Figure 3) This observation was further supported by our finding that depletion of KIF5B almost completely inhibited the accumulation of phosphotau and reversed tau-related memory impairment in P301S tau mice.

Our study on the *in vivo* heterozygous knockout of KIF5B in the P301S mouse model of tauopathy showed that a 50% drop in the KIF5B level was sufficient to reduce overall tau levels and rescue the phenotype. Although locomotor deficits of homozygous P301S tau mice were reported at the age of 5 months, no motor dysfunction was found in the P301S^+/−^ mice based on the rotarod experiment, and backcrossing these mice to a WT mice background might have resulted in a delayed phenotypic onset. We did not allow these mice to grow further as our main aim was to assess the memory function in P301S^+/−^. Memory deficits in the homozygous P301S were previously found to occur from 2.5 months of age (Xu et al., 2014), and the memory function of P301S has never been evaluated due to severe motor impairment at 5 months of age. Since we did not find motor impairments in P301S^+/−^ at 8 months of age, we performed auditory-cued fear conditioning to test associative fear memory. The freezing response of the P301S^+/−^ mice was lesser than that of the WT mice to the conditioned stimulus (CS, auditory cue), but P301S^+/−^, KIF5B^+/−^ mice showed a significant increase in the freezing response than the P301S^+/−^ mice to CS during the recall test 24 h after being conditioned to fear. The absence of significant deficits in heterozygous P301S^+/−^,KIF5B^+/−^ mice in these memory tests suggested the neuroprotective role of KIF5B in memory formation and retrieval. The age-related and AD-related episodic memory loss followed a signature pattern of tau pathology, which initially affected the trans entorhinal cortex and the transition zone between the lateral entorhinal cortex and the perirhinal cortex (Braak and Braak, 1997; Maass et al., 2018). The anterior temporal network might be particularly vulnerable to tau deposition in the first phases of the transition from “normal aging” to AD (Cope et al., 2018; Franzmeier et al., 2019). The KIF5B KO-mediated decrease in memory deficits was associated not only with a significant reduction in the overall number of AT8-positive neurons but also with an area-wise specific reduction in the number of AT8-positive neurons in the anterior, medial, and posterior regions of the brains of P301S mice (Figure 4). The results of the biochemical analysis showed that the AT8-positive -tau in P301S^+/−^,KIF5B^+/−^ mice decreased due to a decrease in sarkosyl-insoluble and sarkosyl-soluble protein fractions. This was accompanied by a decrease in AT180, AT00, PHF1, and total tau (HT7) in the sarkosyl-insoluble fraction. These findings were impressive, given that human tau is expressed at five times the levels of endogenous mouse tau in the P301S mouse model (Allen et al., 2002). Since we found that LC3B-II levels were higher in the P301S^+/−^, KIF5B^+/−^ mice compared to that in the P301S^+/−^ mice (Figures 1A,B), the reduction of tau in P301S^+/−^, KIF5B^+/−^ mice might be *via* autophagy-mediated pathways. Although autophagosomes in cortical neurons are known to undergo dynein-dependent unidirectional retrograde transport along the axon (Lee et al., 2011; Maday et al., 2012), Maday et al. (2012) found that both dynein and kinesin remain bound to the axonal autophagosomes. Thus, autophagosomes must undergo stringent regulation during their retrograde axonal trafficking to maximize dynein motor activity while minimizing the activity of related kinesins. Because KIF5B was reduced or eliminated in P301S^+/−^,KIF5B^+/−^mice or HAP1-KO cells respectively, which may have caused an increase in dynein-mediated autophagosome trafficking, resulting in autophagy-mediated tau degradation in the cell stoma (Figures 3A, 6).

Although there are no tau mutations in AD, the P301S tau transgenic mice are considered to be the best model for AD because they show strong signs of tauopathy (Allen et al., 2002). Since the P301S and P301L mutations disrupt axonal transport and might affect the binding of kinesin to tau (Kneynsberg et al., 2017; Götz, et al., 2019), we hypothesized that KIF5B might control P301S or P301L in the same manner as WT tau. Thus, even a minor decrease in KIF5B might significantly affect tau levels. These findings showed for the first time that KIF5B deficiency significantly affects the tauopathy phenotype. These results might reveal the potential role of KIF5B in the pathogenesis of AD and other tauopathies. Our findings showed that the loss of KIF5B influences the tauopathy phenotype in AD, although it is unclear if restoring the function of KIF5B would have a similar effect. Although our results showed that reducing KIF5B has a neuroprotective effect, the link between kinesin-1 reduction and the neuroprotection caused by a reduction of tau-related phenotypes remains to be studied. Due to the embryonic lethality of KIF5B null animals (Tanaka et al., 1998), the information required to determine the involvement of KIF5B in memory and during neural development *in vivo* is lacking. Zhao et al. (2020) reported abnormalities in dendritic spine development, synaptic plasticity, and memory formation only in homozygous KIF5B conditional knockout mice. Several *in vitro* studies have shown that KIF5B affects neural development; however, we found heterozygous KIF5B KO (50%) mice to be robust and viable without neuroanatomical defects (Lin et al., 2019). The inhibition of kinesin-1 is a viable strategy for treating axonopathy, and various small compounds have been identified (Randall et al., 2017). For example, Randall et al. (2017) identified a small molecule called kinesore, which blocks kinesin-1 from interacting with organelle-specific cargo adaptors without influencing the function of kinesin-1. Our results, along with those reported in previously published studies, suggested that targeting intracellular transport, particularly kinesin-1-associated transport, might delay neurodegeneration. Thus, KIF5B/kinesin-1 is a promising target for the therapeutic intervention of AD and other tauopathies.

Further studies are required on the relationship between tau and KIF5B, as well as its role in identifying the pathways by which KIF5B contributes to neurodegeneration. Understanding how KIF5B works in neurons and astrocytes might help researchers find new ways to treat neurodegeneration.

## Data Availability

The original contributions presented in the study are included in the article/Supplementary Material, further inquiries can be directed to the corresponding authors.
