# Supplementary figures and images for: Reduction of kinesin I heavy chain decreases tau hyperphosphorylation, aggregation, and memory impairment in Alzheimer’s disease and tauopathy models
Source: Front Mol Biosci. 2022 Oct 25;9:1050768. doi: 10.3389/fmolb.2022.1050768 (PMC9641281; doi:10.3389/fmolb.2022.1050768)

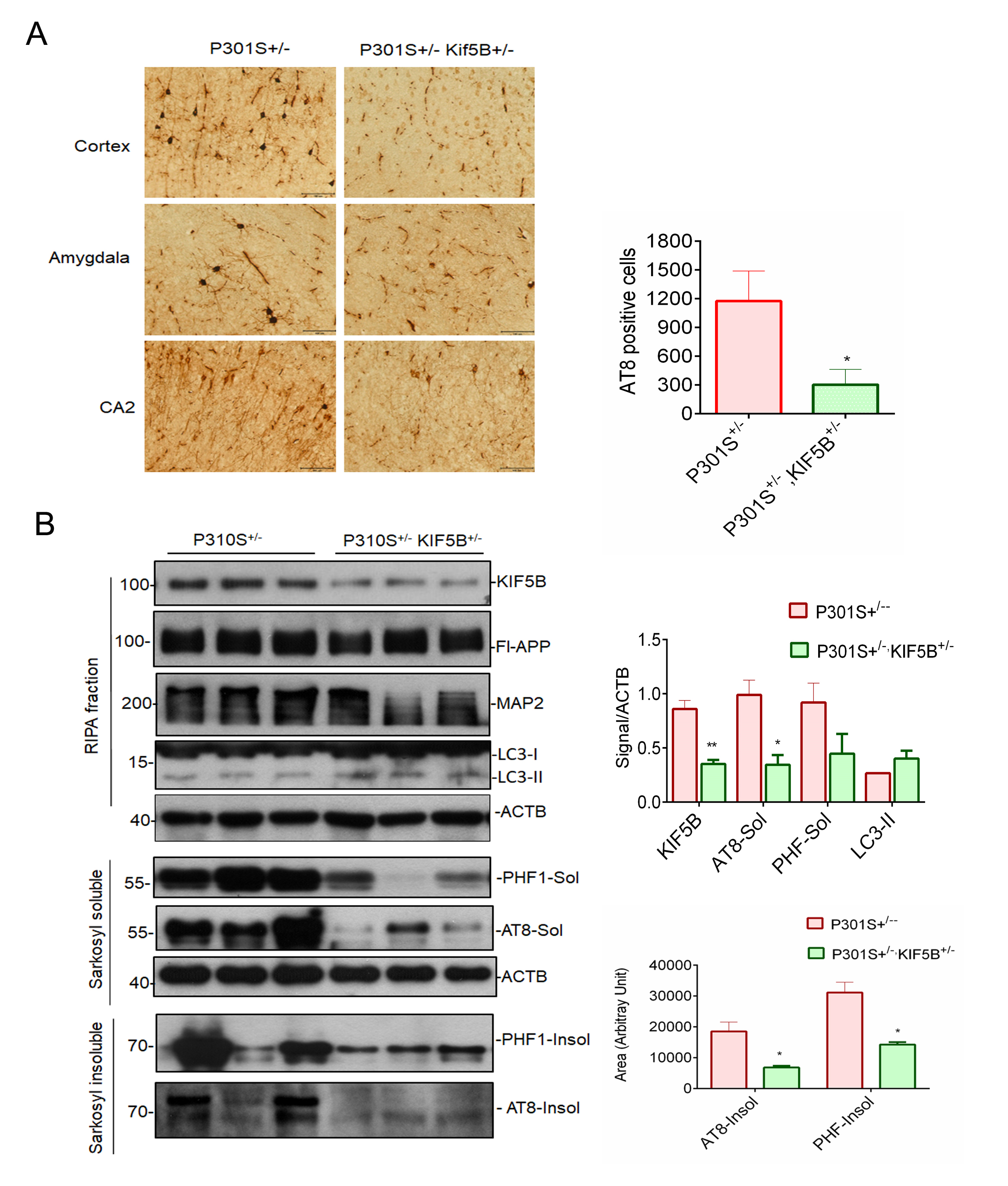

Supplement: Supplementary file 1 [file Image1.jpg]
